# Distinguishing Body Lice from Head Lice by Multiplex Real-Time PCR Analysis of the Phum_PHUM540560 Gene

**DOI:** 10.1371/journal.pone.0058088

**Published:** 2013-02-28

**Authors:** Rezak Drali, Amina Boutellis, Didier Raoult, Jean Marc Rolain, Philippe Brouqui

**Affiliations:** 1 Aix Marseille Université, URMITE, UM63, CNRS 7278, IRD 198, Inserm 1095, Marseille, France; 2 Service des Entérobactéries et Hygiène de l'Environnement, Institut Pasteur d'Algérie, Algiers, Algeria; Cairo University, Egypt

## Abstract

**Background:**

Body louse or head louse? Once removed from their environment, body and head lice are indistinguishable. Neither the morphological criteria used since the mid-18th century nor the various genetic studies conducted since the advent of molecular biology tools have allowed body lice and head lice to be differentiated. In this work, using a portion of the Phum_PHUM540560 gene from the body louse, we aimed to develop a multiplex real-time polymerase chain reaction (PCR) assay to differentiate between body and head lice in a single reaction.

**Materials and Methods:**

A total of 142 human lice were collected from mono-infested hosts from 13 countries on five continents. We first identified the louse clade using a cytochrome b (CYTB) PCR sequence alignment. We then aligned a fragment of the Phum_PHUM540560 gene amplified from head and body lice to design-specific TaqMan^©^ FAM- and VIC-labeled probes.

**Results:**

All the analyzed lice were Clade A lice. A total of 22 polymorphisms between the body and head lice were characterized. The multiplex real-time PCR analysis enabled the body and head lice to be distinguished in two hours. This method is simple, with 100% specificity and sensitivity.

**Conclusions:**

We confirmed that the Phum_PHUM540560 gene is a useful genetic marker for the study of lice.

## Introduction

Body and head lice are hematophagous ectoparasites that are specific to humans [1] and have different ecologies. The body louse, *Pediculus humanus corporis*, lives and multiplies in clothing, whereas the head louse, *Pediculus humanus capitis*, lives and lays its eggs on hair [2], [3]. The body louse is known as a vector of three life-threatening infectious diseases: epidemic typhus, caused by *Rickettsia prowazekii*; relapsing fever, caused by *Borrelia recurrentis*; and trench fever, caused by *Bartonella quintana* [4], [5].

Distinguishing body from head lice has always been a challenge. Once a louse leaves its biotope (head or clothes), it becomes indistinguishable from other lice, which has presented a critical problem in historical and paleobiological studies of lice.

Since the mid-18th century, morphological criteria such as size, shape and color gradation have been used to differentiate body and head lice into two distinct species [6]. In 1978, the use of microscopes to observe body and head lice collected from Ethiopians with double infestations allowed a researcher to conclude that the lice represented two distinct species, *Pediculus humanus Linnaeus* and *Pediculus capitis De Geer*. He based his assertion on the length of the tibia of the louse's middle leg [7].

The advent of molecular biology and gene sequencing has led to the development of genetic studies to address issues concerning louse phylogeny. The investigation of the gene that encodes the 18S ribosomal RNA has enabled the sub-Saharan African phylogenetic group of lice to be distinguished from a second group that encompasses the remainder of the lice worldwide [8], [9]. An analyses of the mitochondrial cytochrome b (CYTB) and cytochrome oxidase I (COI) genes, have allowed the differentiation of three clades of lice. Clade A contains both body and head lice that are distributed worldwide. Clade B contains head lice encountered in America, Europe and Australia, whereas Clade C contains head lice found in Ethiopia, Nepal and Senegal [10]–[14]. Recently, a method targeting intergenic spacers that utilizes four highly polymorphic markers has revealed associations between the sources and genotypic distributions of lice [15], [16]. Nevertheless, none of the above genetic studies were able to differentiate between body and head lice. In 2010, the sequencing of the entire genome of *P. humanus corporis* provided new perspectives for understanding the relationship between the biology and genetics of the louse [17]. More recently, a study comparing the transcriptional profiles of body and head lice reported that the two types of lice had a single, 752-base pair (bp) difference in the Phum_PHUM540560 gene, which encodes a hypothetical, 69-amino acids protein of unknown function [18]. Based on the alignment of a portion of the two Phum_PHUM540560 gene sequences, we have designed a novel multiplex real-time PCR assay to efficiently differentiate, for the first time, between body and head lice collected from a mono-infested host. This assay has been tested by analyzing a large collection of worldwide specimens belonging to Clade A, the only clade known to contain both body and head lice.

## Materials and Methods

### Ethics statement

Lice from foreign countries were obtained from the private frozen collection of our laboratory (The URMITE/WHO Collaborative Research Center). The lice in that collection were required for various epidemiological and entomological studies or to perform diagnoses abroad and were sent to our laboratory as a WHO reference facility. The specimens were collected according to the ethics laws of each country; however, because lice are not part of the human body, lice removed from individuals are not considered to be human samples in most countries. The body lice were collected from clothing, and the head lice were removed from hair, with the verbal consent of the infested individuals. Written consent was not obtainable in the majority of cases because most of the subjects were illiterate. However, in most instances, the investigator, local authorities and/or village chief approved and were present when it was performed.

The lice collected in France were obtained from homeless individuals during a registered epidemiological study (French Bioethics laws n° 2011–814). Informed consent was obtained from these subjects, and the study was approved by the “Comité de Protection des Personnes Sud Mediterranée I” on January, 12, 2011 (ID RCB: 2010-A01406-33).

The anonymity of the individuals who provided the lice used in the present genetic analysis was preserved.

### Sampling

A total of 142 lice, including 88 body lice and 54 head lice, were collected from mono-infested human hosts. The head lice were collected exclusively from the hair, and the body lice were collected exclusively from clothing. No lice were collected from the neck or the beard; the purpose of this precaution was the avoidance hybrid lice, as previously reported [7]. The strain information, geographic origin and anatomical sources (body or head) of the analyzed lice are provided in Table 1.

**Table 1:** The Clade A lice examined in this study and the results of the real-time PCR assay.

Country	Town/province	Analysis channel results		
		Number	FAM-positive	VIC-positive
**Body lice**				
France	Marseille	15	15	0
Hungary	Budapest	10	10	0
Nepal	Pokava	9	9	0
China	Inner Mongolia Province	5	5	0
	Tiligi	7	7	0
Japan	Tokyo	10	10	0
Madagascar	Borenty village	9	9	0
Kenya	Nairobi	10	10	0
USA	Orlando	13	13	0
**Head lice**				
USA	Washington	6	0	6
Brazil	Sao Cristovao	6	0	6
	Amazonia	8	0	8
Madagascar	Bedaro village	12	0	12
Senegal	Dakar	3	0	3
Australia	Brisbane	5	0	5
Papua New Guinea	Highlands	5	0	5
New Zeland	Auckland	9	0	9

### DNA preparation

Prior to DNA isolation, each louse was immersed in 70% ethanol for 15 min and was then rinsed twice in sterile water. Total genomic DNA was extracted using the QIAamp Tissue Kit (QIAGEN, Hilden, Germany) according to the manufacturer's instructions. The extracted DNA was assessed for quantity and quality using a NanoDrop instrument (Thermo Scientific, Wilmington, United Kingdom) before being stored at −20°C [19].

### Conventional PCR and sequencing

Two conventional PCR experiments were performed in this study. The first was performed to identify the Clades of the collected lice by amplifying and sequencing a 347-bp fragment of the mitochondrial cytochrome b (CYTB) gene [7]. The second PCR targeted a 187-bp fragment of the Phum_ PHUM540560 gene using a pair of primers designed in this study and based on the Phum_PHUM540560 gene sequence available from GenBank (*Pediculus humanus corporis* strain USDA 1103172108290, GenBank accession no. NW_002987859.1 GI: 242022583). The obtained PCR products from three body lice and three head lice were sequenced to enable comparison of the body and head lice DNA sequences. All the PCRs were performed using the primers outlined in Table 2 and a PTC-200 automated thermal cycler (MJ Research, Waltham, MA, USA). The final reaction volume was 20 µl, with 0.4 U of Phusion polymerase (Finnzymes, Thermo Scientific, Vantaa, Finland), 4 µl of 5x Phusion buffer, 0.5 mM of each primer, 0.16 mM dNTP mix and 30–50 ng of genomic DNA. The following cycling conditions were used for the amplifications: an initial 30-s denaturation at 98°C; 35 cycles of denaturation for 5 s at 98°C and annealing for 30 s at 56°C (for CYTB gene) or 59°C (for the Phum_PHUM540560 gene); and a final 15 min extension at 72°C. The amplification was completed by a 5-min extension at 72°C. Subsequently, the PCR products were subjected to electrophoresis on 1.5% agarose gels with ethidium bromide staining and were then purified using NucleoFast 96 PCR Plates (Macherey-Nagel EURL, Hoerdt, France) according to the manufacturer's instructions.

**Table 2:** The oligonucleotide primers and probes used in this study.

Name	Purpose	Sequence 5′→3′
*Cytb*_F	Forward sequencing primer partial cytochrome b gene	GAGCGACTGTAATTACTAATC
*Cytb*_R	Reverse sequencing primer partial cytochrome b gene	GGACCCGGATAATTTTGTTG
Phum540560_F	Forward sequencing primer partial Phum_PHUM540560 gene	GTCACGTTCGACAAATGTT
Phum540560_R	Reverse sequencing primer partial Phum_PHUM540560 gene	TTTCTATAACCACGACACGATAAAT
BL probe	Specific to the body lice	**FAM**-CGATCACTCGAGTGAATTGCCA-**TAMRA**
HL probe	Specific to the head lice	**VIC**-CTCTTGAATCGACGACCATTCGCT-**TAMRA**

Bidirectional DNA sequencing of the targeted PCR products was performed using the 3130XL genetic analyzer (Applied Biosystems, Courtaboeuf, France) with the BigDye Terminator v1.1 cycle (Applied Biosystems). The electropherograms obtained for each sequence were analyzed using Chromas Pro software (Technelysium PTY, Australia).

### Phylogenetic analysis

The DNA sequences were aligned using the multi-sequence alignment software CLUSTAL X, version 2.0.11. The partial CYTB gene sequences were aligned with sequences available from GenBank. The percentages of similarity were determined using the MEGA 5 software package (Molecular Evolution Genetic Analysis, The Biodesign Institute, AZ, USA) [20]. The PhyML phylogeny software was used to create an unrooted phylogenetic tree based on the DNA sequences using maximum likelihood (ML) 100 bootstrap replicates [21].

### Real-time PCR and PCR products sequencing

TaqMan^©^ FAM- and VIC-labeled probes (Table 2) specific to body and head lice, respectively, were designed for the sequences obtained in this study. Both probes contained a TAMRA quencher dye at the 3′ end. The probes were synthesized by Applied Biosystems (Courtaboeuf, France).

Monoplex and multiplex real-time PCRs were performed in the CFX96 thermal cycler (Bio-Rad Laboratories, Foster City, CA, USA). The final reaction volume of 20 µl contained 5–20 ng of the DNA template, 10 µl of 2x QuantiTect Probe PCR Master Mix (Qiagen), 0.5 µM of each primer and 0.2 µM of the FAM- or VIC-labeled probes. A monoplex protocol designed to optimize the conditions for the multiplex real-time PCR was used: a denaturation step at 95°C for 15 min; and 40 cycles of 95°C for 15 s and 60°C for 45 s. The multiplex real-time PCR was performed using the optimized conditions that were determined in the monoplex real-time PCR assay. Each reaction contained 10 µl of 2x QuantiTect Probe PCR Master Mix (Qiagen), 0.5 µM of each primer, 0.2 µM of each fluorogenic probe, 5–20 ng of the DNA template, adjusted to a final volume of 20 µl with the addition of nuclease-free dH_2_O. The cycling parameters consisted of 95°C for 15 min and 40 cycles of 95°C for 15 s and 60°C for 1 min. To evaluate the specificity (the ability of the test to identify negative results) and sensitivity (the ability of the test to identify positive results) of the developed method, all the products of the multiplex real-time PCR amplifications were sequenced, and these sequences were used as the gold standard reference.

## Results

The concentration of the genomic DNA extracted from the 142 lice analyzed in this study ranged from 5 to 20 ng/µl.

### Genotypic distribution of the lice based on the mitochondrial CYTB gene

A 347-bp DNA fragment was successfully amplified from the CYTB gene in all 142 lice. Direct sequencing and multiple alignments of the obtained sequences revealed that all the lice belonged to Clade A (data not shown).

### Characterization of the partial Phum_PHUM540560 gene in body and head lice

**Figure 1:**
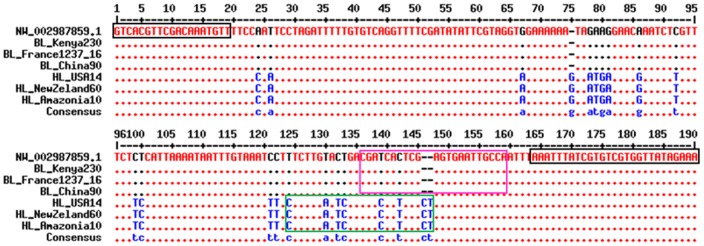
Primer and probe alignments with partial Phum_PHUM540560 gene sequences from body and head lice [35]. A portion of the Phum_PHUM540560 gene sequences from body and head lice were aligned with the primers and probes designed for the multiplex RT-PCR assay. Part of the first exon spanings nucleotides 1 to 64 was analyzed. The forward and reverse primer sequences are boxed in black. The FAM- and VIC-labeled probe sequences are boxed in purple and green, respectively. The nucleotides in blue represent single-nucleotide polymorphisms that are specific to head lice. The nucleotides in black represent single-nucleotide polymorphisms that are specific to body lice. BL: body louse; HL: head louse; NW_002987859.1: *Pediculus humanus corporis* strain USDA 1103172108290 Phum_PHUM540560 (gene sequence available in GenBank).

The multiple alignments of the partial Phum_PHUM54056 gene sequences obtained from the six analyzed lice revealed 22 polymorphisms between body and head lice (Figure 1). The first exon contained two point mutations: a silent (CCA>CCC) transversion affecting codon 18 that would not change the amino acid and the I19N (ATT>AAT) mutation that would replace isoleucine with asparagine.

The remainder of the polymorphisms were spread throughout the first intron and included the insertion of nucleotides at two different locations: approximately nucleotide (nt) 96+11 ins.G and nt 96+80, (ins.CT). This triplex insertion resulted in the amplification of a 190-bp fragment from the head lice and a 187-bp fragment from the body lice (Figure 1).

### Real-time PCR and PCR product sequencing

The monoplex real-time PCR results demonstrated that the FAM-labeled probe was specific to the body lice and that the VIC-labeled probe was specific to the head lice. This assay was optimized by testing louse specimens from known anatomical locations.

**Figure 2:**
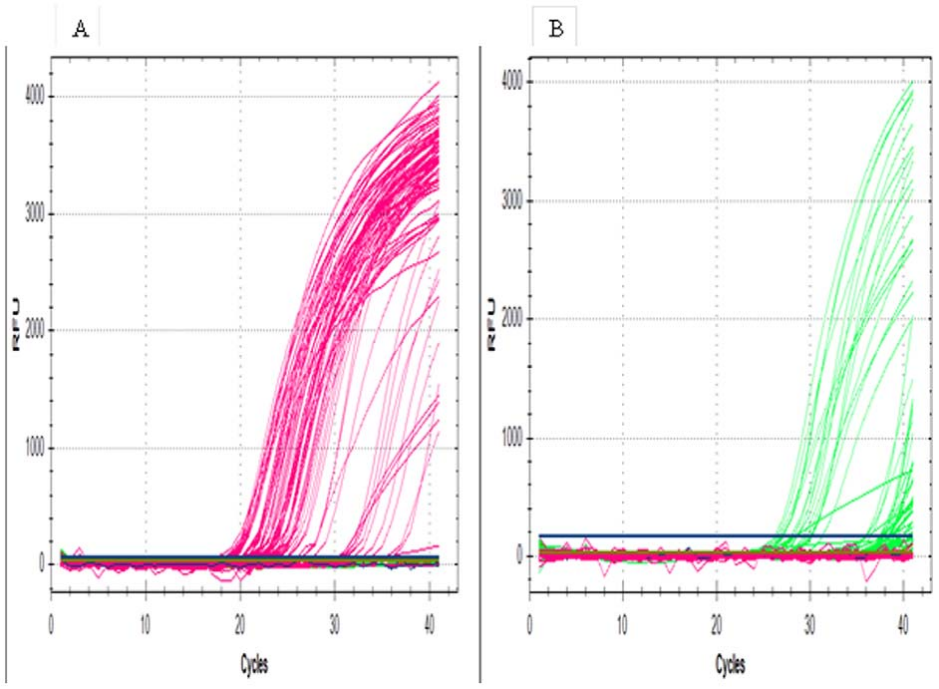
Amplification curves from multiplex real-time PCR assays. Figure 1A. Real-time PCR amplification curves for body lice using a partial Phum_PHUM540560 gene in the FAM channel (495–520). Figure 1B. Amplification curves for head licee louse using a partial Phum_PHUM540560 gene in the VIC channel (522–544).

The multiplex real-time PCR assay clearly identified and simultaneously differentiated among the 142 lice included in this work. Specifically, the signal emitted by the FAM-labeled probe was detected only in the body louse samples, whereas the signal emitted by the VIC-labeled probe was detected only in the head louse samples (Figure 2). No signals were detected in the non-template controls (NTCs). The Ct values obtained in this assay are outlined in Table S1. The sequencing of the 142 PCR products has confirmed our results. In addition, 100% of the samples that were positive for the FAM-labeled probe contained sequences specific to body lice, and 100% of the samples that were positive for the VIC-labeled probe contained sequences specific to head lice (100% sensitivity and 100% specificity; data not shown).

## Discussion

Presently, comparisons of the body and head lice genomes are not possible because the head louse genome is not yet available. Recently published comparative transcriptional profiles of both body and head lice demonstrated that among the nine genes with differential expression, only one gene was absent in the head louse but present in the body louse [18]. We considered this difference to be a possible opportunity for distinguishing body lice from head lice. Unexpectedly, our first PCR amplification of the 187- bp fragment of the Phum_PHUM540560 gene produced a PCR product from both head and body louse samples, suggesting that at least a portion of the gene was present in both types of lice. The sequencing of the PCR products revealed significant differences between the sequences from the head and body lice, which may explain why the head louse sequence failed to amplify in Old's experiment [18].

In this study, we exploited this sequence variation in the partial Phum_PHUM540560 gene to discriminate between body and head lice from a global collection of lice collected from mono-infested hosts originating from five different continents. Lice from Clade A were used because Clade A is the only currently recognized clade that includes both head and body lice [10], [13], [14], the two types of lice that our assay was developed to distinguish. Finally, our choice of specimens was based on the commonly recognized definitions of body and head lice. Under these conditions, we developed a multiplex real-time PCR assay that is rapid (two hours) and simple and has 100% specificity and sensitivity.

The purpose of this study was to distinguish between body and head lice, a long-standing challenge. Resolving this challenge has become even more important because both head and body lice have been reported to harbor *Bartonella quintana*, the trench fever agent, raising the question of whether head lice, similar to body lice, can transmit the agent [22], [23]. Currently, *B. quintana* DNA has been detected only in head lice collected from impoverished people in situations where co-infestations with body lice are possible [24]–[27]. In fact, co-infestations have been recently reported in the same homeless population [28]. One study of head lice collected from schoolchildren in France failed to detect *B. quintana* [29]. The ability to distinguish body lice from head lice will help advance our understanding of the role of head louse in the transmission of *B. quintana*. Moreover, 22% of the homeless people who frequent shelters in Marseille, France are infested with lice, and some people can harbor more than 10,000 lice in their clothing. In such a situation, finding lice on the head challenges the “head louse definition”, making an identification tool useful.

Recent studies have suggested that head and body lice can be mixed in people infested with both types of lice [28]. Although head and body lice do not interbreed in the wild [7], fertile hybrids with an intermediate morphology [30] have been reported under laboratory conditions [31], [32]. Moreover, several observational studies have also suggested that head lice may become body lice when raised under the appropriate conditions [33], [34]. Our technique can be used to identify heterozygous specimens, which may prove valuable for studies on the population dynamics of lice.

This work confirmed that the Phum_PHUM540560 gene may be a useful genetic marker for the study of lice. However, the genetic differences between head and body lice do not put back into question whether head and body lice are conspecific [11]. The ability to distinguish between head and body lice may facilitate future research into the behavior of Clade A body and head lice.

## Supporting Information

Table S1 Ct values obtained in multiplex real-time PCR for differentiating between body and head louse.
(DOCX)
