# Correction: Distinguishing Body Lice from Head Lice by Multiplex Real-Time PCR Analysis of the Phum_PHUM540560 Gene

**DOI:** 10.1371/journal.pone.0311640

**Published:** 2024-10-01

**Authors:** The PLOS ONE Editors

This Correction resolves the prior Expression of Concern for the linked article [1, 2].

Following the publication of the article and Expression of Concern [1, 2], PLOS investigated the concerns pertaining to the reported ethical oversight and the article’s adherence to PLOS research ethics policies.

Specifically, PLOS found that the ethics approval number was also reported in multiple other publications [3–20], despite apparent differences in the study objectives, periods during which the data were collected, sample sizes, sampling methodology and locations, types of samples being collected, and types of consent. Furthermore, the *PLOS ONE* article [1] does not state when samples were collected.

A representative from the Aix-Marseille Université stated that the institutional investigation into the ethics concerns concluded this article meets ethical standards. They stated that no invasive or risky sampling was carried out for this study, and they indicated that collection of lice would not require ethics approval.

The study reported in [1] included lice collected in two contexts: (i) in a CPP-approved study that involved a treatment (CPP approval RCB: 2010-A01406-33), and (ii) from people in foreign countries.

Approval #2010-A01406-33 was issued by the Comité de Protection des Personnes Sud Méditerranée I on or after 24 January 2011, and grants approval for a study titled “*Eradication du portage du pou de corps par vetements impregnes de perméthrine*: *essai comparatif randomise versus placebo en double aveugle dans les populations défavorisées de Marseille*”. The ethics document approves a randomized comparative trial, whereas the Materials and Methods section of this article [1] states that the lice collected in France were obtained during a registered epidemiological study. The representative of the Aix-Marseille Université Ethics Committee confirmed that the statement in [1] is incorrect and that the French samples used in [1] were collected during a clinical trial [5]. They further stated that [1] was one of several secondary studies that stemmed from the trial and would therefore report the same ethics approval number. The article reporting the clinical trial [5] states that participants were recruited in February 2011 and December 2011.

In light of the information provided by the institute, the second paragraph of the Ethics statement in the Materials and Methods section is updated to:

The lice collected in France were obtained from homeless individuals during a randomized comparative trial involving a study into the eradication of lice by clothing impregnated with permethrin (reference [5] of this Correction notice; clinicaltrials.gov # NCT01287663). Informed consent was obtained from participants, and the randomized comparative trial was approved by the “Comité de Protection des Personnes Sud Mediterranée I” on January, 24, 2011 (ID RCB: 2010-A01406-33).

According to the Methods section of this article [1], “*Lice from foreign countries were obtained from the private frozen collection of our laboratory (The URMITE/WHO Collaborative Research Center)*. *The lice in that collection were required for various epidemiological and entomological studies or to perform diagnoses abroad and were sent to our laboratory as a WHO reference facility*. *The specimens were collected according to the ethics laws of each country*”.

PLOS has not received any ethics documentation or research authorizations to confirm the reported ethics information about lice collected outside the CPP-approved study. Nevertheless, based on the information currently available the *PLOS ONE* Editors are satisfied that [1] met the journal’s research ethics requirements since it was a secondary analysis of samples from a WHO reference facility and a CPP-approved study.

With this update, the *PLOS ONE* Editors consider the ethics approval concerns resolved. This Correction supersedes the prior Expression of Concern [2].
