# Supplementary material for: Distinguishing Body Lice from Head Lice by Multiplex Real-Time PCR Analysis of the Phum_PHUM540560 Gene
Source: PLoS One. 2013 Feb 28;8(2):e58088. doi: 10.1371/journal.pone.0058088 (PMC3585238; doi:10.1371/journal.pone.0058088)
Supplement: Table S1 — Ct values obtained in multiplex real-time PCR for differentiating between body and head louse. (DOCX) [file pone.0058088.s001.docx]

**Table S1.** Ct values obtained in multiplex real-time PCR for differentiating between body and head louse

| **Louse** | **Ct values** | | **Country** | **Town/Province** |
| --- | --- | --- | --- | --- |
|  | **FAM** | **VIC** |  |  |
| Body louse_1003/4 | 21,56 | NA | France | Marseille |
| Body louse_1160/1 | 20,84 | NA |  |  |
| Body louse_1200/2 | 20,02 | NA |  |  |
| Body louse_1229/6 | 18,33 | NA |  |  |
| Body louse_1237/9 | 19,04 | NA |  |  |
| Body louse_1237/10 | 20,19 | NA |  |  |
| Body louse_1237/11 | 20,11 | NA |  |  |
| Body louse_1237/16 | 20,24 | NA |  |  |
| Body louse_1237/17 | 20,09 | NA |  |  |
| Body louse_1237/19 | 20,51 | NA |  |  |
| Body louse_1240/1 | 20,87 | NA |  |  |
| Body louse_1245/4 | 20,72 | NA |  |  |
| Body louse_1246/1 | 18,74 | NA |  |  |
| Body louse_2002/2 | 19,04 | NA |  |  |
| Body louse_2215 | 20,10 | NA |  |  |
| Body louse_104 | 22,02 | NA | Hungary | Budapest |
| Body louse_105 | 25,10 | NA |  |  |
| Body louse_106 | 26,51 | NA |  |  |
| Body louse_107 | 23,90 | NA |  |  |
| Body louse_108 | 24,17 | NA |  |  |
| Body louse_109 | 22,10 | NA |  |  |
| Body louse_110 | 27,59 | NA |  |  |
| Body louse_111 | 22,74 | NA |  |  |
| Body louse_112 | 23,19 | NA |  |  |
| Body louse_113 | 25,00 | NA |  |  |
| Body louse_80 | 23,08 | NA | China | Inner Mongolia Province |
| Body louse_82 | 21,58 | NA |  |  |
| Body louse_83 | 23,50 | NA |  |  |
| Body louse_83 | 30,84 | NA |  |  |
| Body louse_86 | 31,98 | NA |  |  |
| Body louse_87 | 20,68 | NA |  | Tiligi |
| Body louse_88 | 22,17 | NA |  |  |
| Body louse_89 | 26,41 | NA |  |  |
| Body louse_90 | 20,49 | NA |  |  |
| Body louse_91 | 22,43 | NA |  |  |
| Body louse_92 | 21,94 | NA |  |  |
| Body louse_93 | 22,84 | NA |  |  |
| Body louse_94 | 21,74 | NA | Japan | Tokyo |
| Body louse_95 | 21,28 | NA |  |  |
| Body louse_96 | 20,08 | NA |  |  |
| Body louse_97 | 20,89 | NA |  |  |
| Body louse_98 | 21,43 | NA |  |  |
| Body louse_99 | 23,07 | NA |  |  |
| Body louse_100 | 21,86 | NA |  |  |
| Body louse_101 | 22,04 | NA |  |  |
| Body louse_102 | 22,07 | NA |  |  |
| Body louse_103 | 22,42 | NA |  |  |
| Body louse_70 | 21,66 | NA | Nepal | Pokava |
| Body louse_71 | 21,71 | NA |  |  |
| Body louse_72 | 23,48 | NA |  |  |
| Body louse_73 | 23,08 | NA |  |  |
| Body louse_74 | 22,29 | NA |  |  |
| Body louse_75 | 23,22 | NA |  |  |
| Body louse_76 | 26,08 | NA |  |  |
| Body louse_78 | 24,11 | NA |  |  |
| Body louse_79 | 23,88 | NA |  |  |
| Body louse_223 | 30,34 | NA | Kenya | Nairobi |
| Body louse_224 | 35,20 | NA |  |  |
| Body louse_226 | 33,64 | NA |  |  |
| Body louse_227 | 30,26 | NA |  |  |
| Body louse_228 | 36,03 | NA |  |  |
| Body louse_229 | 30,68 | NA |  |  |
| Body louse_230 | 31,23 | NA |  |  |
| Body louse_231 | 30,21 | NA |  |  |
| Body louse_232 | 32,72 | NA |  |  |
| Body louse_233 | 35,21 | NA |  |  |
| Body louse_175 | 20,46 | NA | Madagascar | Borenty village |
| Body louse_176 | 21,04 | NA |  |  |
| Body louse_177 | 20,10 | NA |  |  |
| Body louse_178 | 20,12 | NA |  |  |
| Body louse_179 | 22,11 | NA |  |  |
| Body louse_180 | 21,87 | NA |  |  |
| Body louse_181 | 23,76 | NA |  |  |
| Body louse_182 | 23,45 | NA |  |  |
| Body louse_183 | 23,02 | NA |  |  |
| Body louse_PDL1 | 19,28 | NA | USA | Laboratory colony |
| Body louse_PDL3 | 18,88 | NA |  |  |
| Body louse_PDL4 | 18,66 | NA |  |  |
| Body louse_PDL6 | 18,62 | NA |  |  |
| Body louse_PDL11 | 18,90 | NA |  |  |
| Body louse_PDL13 | 18,57 | NA |  |  |
| Body louse_PDL14 | 19,13 | NA |  |  |
| Body louse_PDL7 | 18,92 | NA |  |  |
| Body louse_PDL2 | 18,30 | NA |  |  |
| Body louse_PDL9 | 17,53 | NA |  |  |
| Body louse_PDL10 | 19,01 | NA |  |  |
| Body louse_PDL4 | 21,18 | NA |  |  |
| Body louse_PDL5 | 21,03 | NA |  |  |
| Head louse_13 | NA | 31,52 | USA | Washington |
| Head louse_14 | NA | 32,36 |  |  |
| Head louse_15 | NA | 32,04 |  |  |
| Head louse_16 | NA | 35,44 |  |  |
| Head louse_17 | NA | 37,34 |  |  |
| Head louse_19 | NA | 36,01 |  |  |
| Head louse_9 | NA | 34,43 | Brazil | Amazonia |
| Head louse_10 | NA | 35,87 |  |  |
| Head louse_11 | NA | 33,92 |  |  |
| Head louse_12 | NA | 39,17 |  |  |
| Head louse_14 | NA | 35,69 |  |  |
| Head louse_172 | NA | 37,51 |  |  |
| Head louse_173 | NA | 36,56 |  |  |
| Head louse_175 | NA | 37,32 |  |  |
| Head louse_20 | NA | 35,47 |  | Sao Cristovao |
| Head louse_21 | NA | 37,84 |  |  |
| Head louse_23 | NA | 35,22 |  |  |
| Head louse_24 | NA | 39,33 |  |  |
| Head louse_25 | NA | 38,18 |  |  |
| Head louse_29 | NA | 23,20 |  |  |
| Head louse_30 | NA | 36,56 | Australia | Brisbane |
| Head louse_31 | NA | 35,45 |  |  |
| Head louse_34 | NA | 38,16 |  |  |
| Head louse_36 | NA | 37,01 |  |  |
| Head louse_37 | NA | 37,88 |  |  |
| Head louse_50 | NA | 36,43 | Papua New Guinea | Highlands |
| Head louse_51 | NA | 36,33 |  |  |
| Head louse_54 | NA | 36,26 |  |  |
| Head louse_55 | NA | 36,21 |  |  |
| Head louse_56 | NA | 37,64 |  |  |
| Head louse_60 | NA | 35,71 | New Zeeland | Auckland |
| Head louse_61 | NA | 35,94 |  |  |
| Head louse_62 | NA | 37,74 |  |  |
| Head louse_63 | NA | 36,43 |  |  |
| Head louse_64 | NA | 38,07 |  |  |
| Head louse_66 | NA | 37,20 |  |  |
| Head louse_67 | NA | 36,89 |  |  |
| Head louse_68 | NA | 37,45 |  |  |
| Head louse_69 | NA | 37,16 |  |  |
| Head louse_160 | NA | 24,45 | Madagascar | Bedaro village |
| Head louse_161 | NA | 24,79 |  |  |
| Head louse_162 | NA | 24,54 |  |  |
| Head louse_163 | NA | 36,13 |  |  |
| Head louse_165 | NA | 24,09 |  |  |
| Head louse_166 | NA | 28,02 |  |  |
| Head louse_168 | NA | 26,78 |  |  |
| Head louse_170 | NA | 26,23 |  |  |
| Head louse_171 | NA | 36,16 |  |  |
| Head louse_172 | NA | 28,20 |  |  |
| Head louse_173 | NA | 29,55 |  |  |
| Head louse_174 | NA | 27,44 |  |  |
| Head louse_48 | NA | 27,54 | Senegal | Dakar |
| Head louse_95 | NA | 28,17 |  |  |
| Head louse_96 | NA | 24,03 |  |  |
| NTC | NA | NA |  |  |

NA: signal not detected; NTC: non template control
